# Sidestream cigarette smoke effects on cardiovascular responses in conscious rats: involvement of oxidative stress in the fourth cerebral ventricle

**DOI:** 10.1186/1471-2261-12-22

**Published:** 2012-03-30

**Authors:** Vitor E Valenti, Luiz Carlos de Abreu, Monica A Sato, Celso Ferreira, Fernando Adami, Fernando LA Fonseca, Valdelias Xavier, Moacir Godoy, Carlos B Monteiro, Luiz Carlos M Vanderlei, Paulo HN Saldiva

**Affiliations:** 1 Programa de Pós-Graduação em Fisioterapia, Faculdade de Ciências e Tecnologia, Universidade Estadual Paulista, UNESP, Presidente Prudente, SP, Brazil; 2 Departamento de Morfologia e Fisiologia, Faculdade de Medicina do ABC, Santo André, SP, Brazil; 3 Departamento de Cardiologia e Cirurgia Cardiovascular, Faculdade de Medicina de São José do Rio Preto, São José do Rio Preto, São Paulo, Brasil; 4 Departamento de Patologia, Faculdade de Medicina, Universidade de São Paulo, São Paulo, SP, Brazil; 5 Departamento de Fonoaudiologia, Faculdade de Filosofia e Ciências, Universidade Estadual Paulista, UNESP, Av. Higyno Muzzi Filho, 737, Marília, SP 17525-900, Brazil

## Abstract

**Background:**

Cigarette exposure increases brain oxidative stress. The literature showed that increased brain oxidative stress affects cardiovascular regulation. However, no previous study investigated the involvement of brain oxidative stress in animals exposed to cigarette and its relationship with cardiovascular regulation. We aimed to evaluate the effects of central catalase inhibition on baroreflex and cardiovascular responses in rats exposed to sidestream cigarette smoke (SSCS).

**Methods:**

We evaluated males Wistar rats (320-370 g), which were implanted with a stainless steel guide cannula into the fourth cerebral ventricle (4th V). Femoral artery and vein were cannulated for mean arterial pressure (MAP) and heart rate (HR) measurement and drug infusion, respectively. Rats were exposed to SSCS during three weeks, 180 minutes, 5 days/week (CO: 100-300 ppm). Baroreflex was tested with a pressor dose of phenylephrine (PHE, 8 μg/kg, bolus) to induce bradycardic reflex and a depressor dose of sodium nitroprusside (SNP, 50 μg/kg, bolus) to induce tachycardic reflex. Cardiovascular responses were evaluated before, 5, 15, 30 and 60 minutes after 3-amino-1,2,4-triazole (ATZ, catalase inhibitor, 0.001 g/100 μL) injection into the 4th V.

**Results:**

Central catalase inhibition increased basal HR in the control group during the first 5 minutes. SSCS exposure increased basal HR and attenuated bradycardic peak during the first 15 minutes.

**Conclusion:**

We suggest that SSCS exposure affects cardiovascular regulation through its influence on catalase activity.

## Background

Exposure to environmental tobacco smoke is recognized as a significant contributor to cardiovascular mortality. The impact of ambient cigarette smoke on cardiovascular and respiratory systems may be an important factor in the observed adverse cardiovascular health effects [1-3]. Cigarette smoke is classified into two categories, the mainstream smoke usually inhaled by active smokers, and the sidestream smoke emitted from a cigarette and inhaled by so-called "passive smokers". It is known that sidestream cigarette smoke (SSCS) contains a variety of oxidants and other harmful compounds much more than that contained in mainstream smoke [4]. Passive smokers are thus exposed to almost the same chemicals in cigarette smoke as active smokers are. Therefore, passive smoking increases the risk of cardiac or other related disease in nonsmokers [5].

Oxidative stress caused by cigarette smoke occurs due to the direct effects of the radicals present in smoke [6]. Reactive oxygen species (ROS), such as superoxide anions (O_2_^-^) and hydrogen peroxide (H_2_O_2_), are recognized as dangerous second messengers in many mechanisms [7]. ROS are the result of incomplete reduction of oxygen to O_2_^-^which is spontaneously or enzymatically dismutated to H_2_O_2 _by superoxide dismutase (SOD). H_2_O_2 _is transformed to H_2_O and O_2 _under catalase activity [8]. Among the negative effects of ROS we may include lipid peroxidation, which impairs the cell function, while low physiological levels of H_2_O_2 _is able to act as a classical intracellular signalling molecule regulating kinase-driven pathways [9].

The brain influences the cardiovascular system [10,11]. Previous investigations suggested that brain ROS is associated with increased sympathetic activity [12,13] and systemic ROS is also related to impaired baroreflex [14]. Oxidative stress in the rostral ventrolateral medulla (RVLM) is increased and contributes to the neural mechanisms of hypertension in stroke-prone spontaneously hypertensive rats [15,16]. Moreover, activation of the nicotinamide adenine dinucleotide phosphate oxidase through the angiotensin type 1 (AT1) receptors is indicated to be the major source of ROS production, and an altered downstream signaling pathway is involved in the activation of the RVLM neurons, leading to enhanced central sympathetic outflow and hypertension [17].

The activity of sympathetic and parasympathetic systems is under the control of a medullary circuitry comprising the nucleus of the solitary tract (NTS), rostral (RVLM) and caudal ventrolateral medulla (CVLM) and the nucleus ambiguus. Drugs injection into the fourth cerebral ventricle (4th V) may easily reach structures surrounding the ventricular system like the area postrema and there is a preference for parasympathetic system which modulates heart rate, such as nucleus ambiguus, the area postrema of the NTS and dorsal motor nucleus of the vagus [18]. In order to verify if any group of neurons is influenced by a drug in this area, the 4th V is usually used to raise this hypothesis [18].

Cerebral circulation is another issue involved in cardiovascular regulation [19]. Furthermore, ROS may play a role as signalling molecules in the brain circulation under pathological and physiological conditions [19]. Taken together, it leads us to hypothesize a relationship between increased brain ROS production induced by SSCS and cardiovascular regulation.

Luchese et al. [20] demonstrated that acute cigarette smoke exposure increases oxidative stress in the brain, however, it is not yet established if this mechanism influence cardiovascular function. Also, Bonham and colleagues [21] suggested that an upregulation of the substance P system at NTS synapses could contribute to the increased NTS excitability and enhanced reflex responses to lung C-fiber stimulation in pigs exposed to SSCS. Bartoli et al. [22] suggested that increased baroreceptor reflex sensitivity may compensate for particle-induced alterations in blood pressure in dogs. However, it lacks in the literature information regarding whether SSCS exposure affects catalase activity into the brainstem and its relationship with the cardiovascular system. Considering that catalase acitivity is inhibited by 3-Amino-1,2,4-triazole (ATZ), this study was undertaken to investigate the effects of catalase inhibition through ATZ into the 4th V on cardiovascular responses and baroreflex in rats exposed to SSCS.

## Methods

### Animals

We used male Wistar rats (320-370 g) which were kept in the Animal Care Unit of our University. Rats were housed individually in plastic cages under standard laboratory conditions. They were kept under a 12 h light/dark cycle (lights on at 07:00 h) and had free access to food and water. The Institution's Animal Ethics Committee of the School of Medicine of ABC authorized housing conditions and experimental procedures (number 0255/10). Research carried out on humans were in compliance with the Helsinki Declaration. Efforts were made to minimize the number of animals used.

### Sidestream cigarette smoke (SSCS) exposure

The rats (n = 7) were placed in a transparent chamber, with a volume of approximately 95 × 80 × 65 cm^3^, where four rats remained. Environmental conditions were at 23 ± 1°C and 50-60% relative humidity. Smoke carbon monoxide (CO) concentration in the chamber was maintained between 100-300 ppm. Rats were placed in a clear chamber. Cigarettes were put into the chamber in a separated box, which in a small box which prevented the rats from touch the cigarettes. SSCS was produced by burning the cigarettes inside the chamber without filtering, which is the main profile of SSCS. Starting point t0 was set, when CO concentration reached 100 ppm. Burned cigarettes were replaced with new ones and CO concentration between 100-300 ppm. Rats were exposed to SSCS for 180 minutes, five days/week, in a period of time of three weeks in total and exposures were performed in the morning, between 8 a.m. and 12 p.m. Commercial brand cigarettes were used with the following composition: 1.1 mg of nicotine, 14 mg of tar and 15 mg of carbon monoxide. Control animals (n = 8) maintained at the same place under the same conditions as the SSCS group but exposed to fresh air [23,24].

### Surgical preparation

Five days before the experiment (one day after the last SSCS exposure), the rats were anesthetized with ketamine (50 mg/kg i.p.) and xylazine (50 mg/kg i.m.). After scalp anesthesia with 2% lidocaine, the skull was exposed and a stainless steel guide cannulas (26 G) were implanted into the 4th V 1 mm above site injection, using a stereotaxic apparatus (Stoelting, USA). Stereotaxic coordinates for cannula implantation into the 4th V were: AP = -13 mm from the bregma; L = 0 mm from the medial suture, V = -6 mm from the skull. Cannulas were fixed to the skull with dental cement and one metal screw.

One day before the experiments, rats were anesthetized with ketamine (50 mg/kg i.p.) and xylazine (50 mg/kg i.m.) and a catheter was inserted into the abdominal aorta through the femoral artery for blood pressure and heart rate recording. Catheters were made of 4 cm segments of PE-10 polyethylene (Clay Adams, USA) heat bound to a 13 cm segment of PE-50. Catheters were tunneled under the skin and exteriorized at the animal's dorsum [25]. After each surgery, the animals received a single dose of an antibiotic (ampicillin, 100 mg/kg) and a single dose of the analgesic ketorolac (0.6 mg/kg).

### Arterial pressure and heart rate recording

After surgery, the animals were kept in individual cages used in the transport to the experimental room. Animals were allowed 60 minutes to adapt to the conditions of the experimental room such as sound and illumination before starting blood pressure and heart rate recording. The experimental room was acoustically isolated and had constant background noise produced by an air exhauster. At least another 30 minutes period was allowed before beginning experiments. Pulsatile arterial pressure of freely moving animals was recorded using an HP-7754A preamplifier (Hewlett Packard, USA) and an acquisition board (MP100A, Biopac Systems Inc, USA) connected to a computer. Mean arterial pressure (MAP) and heart rate (HR) values were derived from the pulsatile arterial pressure recordings and processed on-line [25].

### Baroreflex evaluation

Baroreflex was activated by intravenous phenylephrine (PHE, 8 μg/kg, bolus) or sodium nitroprusside (SNP, 50 μg/kg, bolus). Baroreflex gain was calculated as the derivation of HR in function of the MAP variation (ΔHR/ΔMAP, maximum changes in MAP and HR). Sympathetic baroreflex gain (SBG) was considered as the ΔHR/ΔMAP ratio in response to i.v. SNP and parasympathetic baroreflex gain (PBG) was considered as the ΔHR/ΔMAP ratio in response to i.v. PHE. We also analyzed bradycardic and tachycardic peak and HR range (the difference between bradycardic and tachycardic peak) [25].

### Injections into the 4th V

Injections into the 4th V were made with 10 μl Hamilton syringes connected by polyethylene tubing (PE-10) to an injector needle. The injector, when completely inserted, protruded 2 mm beyond the tip of the guide cannula. Injections into the 4th V were 1.0 μl for about 5-10 s [26].

### Experimental procedure

Baroreflex and cardiovascular responses were evaluated before (control), 5, 15, 30 and 60 minutes after 3-Amino-1,2,4-triazole (ATZ, catalase inhibitor, 0.001 g/100 μL) or vehicle (n = 7) (0.9% NaCl) injection into the 4th V of conscious rats [26]. Local injection of ATZ is a common method for inducing oxidative stress [26-28].

### Histology

At the end of the experiments, the animals were anesthetized with urethane (1.25 g/kg, i.p.) and 200 nl of 1% Evan's blue dye was injected in the 4th V as a marker of the injection site. The chest was surgically opened, the descending aorta occluded, the right atrium severed and the brain perfused with 10% formalin, through the left ventricle. The brains were post fixed for 24 h at 4°C, and 40 μm sections were cut in a cryostat (model CM 1900, Leica, Germany). This type of section and thickness is able to provide evidence regarding the area reached by the injection reached. Brain sections were stained with 1% neutral red. The actual placement of the injection needles was verified in serial section [26].

### Statistical analysis

The results were reported as means ± standard deviation of means (S.D.M.). In order to compare all variables (basal MAP and HR, bradycardic and tachycardic peak, HR range, SBG, PBG, PHE-induced increase and SNP-induced decrease in MAP, bradycardic and tachycardic reflex) analyses of variance (one way ANOVA) for repeated measures followed by the Tukey post test were applied. We compared variables between before (control), 5, 15, 30 and 60 minutes after ATZ injection into the 4th V in the same rat. Differences were considered significant when the probability of a Type I error was less than 5% (*p *< 0.05).

## Results

### Histology

**Figure 1:**
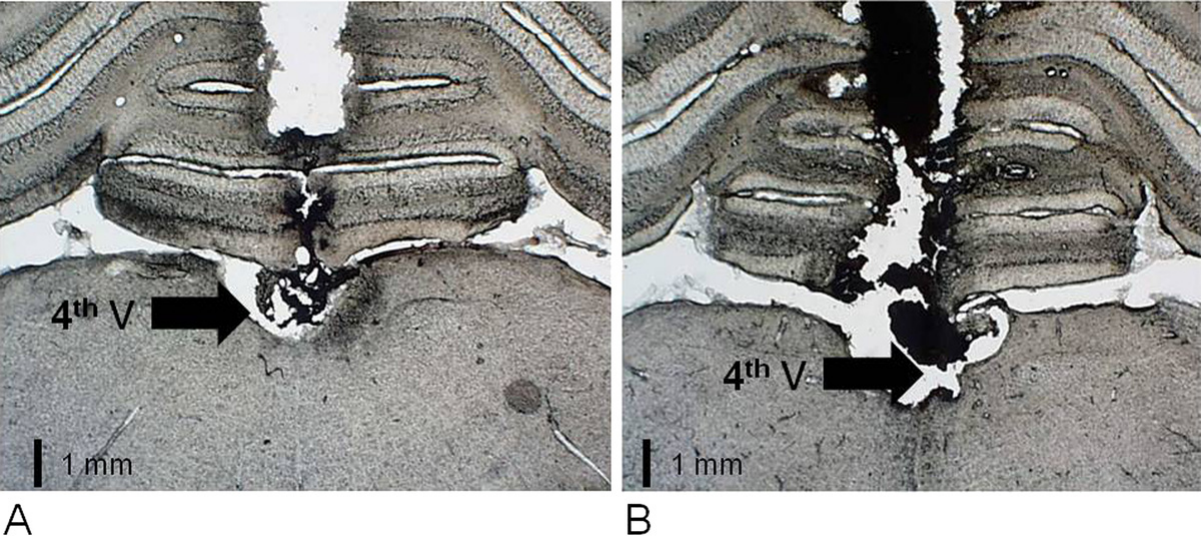
**Photomicrograph of a rat brain showing the medulla and cerebellum (~13 mm caudal to bregma)**. 4th V, fourth ventricle. (**A**) Rat exposed to fresh air, (**B**) rat exposed to SSCS. Schematic draw adapted from Paxinos and Watson. Scale = 1 mm.

Figure 1 is a photomicrograph showing the typical sites of the injections into the 4th V in one rat representative of the rats used in our study. These coronal sections are located approximately 13 mm caudal to bregma.

In order to verify if SSCS exposure could affect the brain of the rats, we compared the photomicrographs between one rat exposed to fresh air (Figure 1A) and another rat exposed to SSCS (Figure 1B). Based on this qualitative analysis, we observed no differences regarding fibrotic tissue around the 4th V between the two groups.

### SSCS effects on basal MAP and HR

There was no difference between rats exposed to fresh air and rats exposed to SSCS regarding basal MAP (Control group: 114 ± 3 mmHg vs. SSCS group: 109 ± 2 mmHg; *p *= 0.78) and HR (Control group: 308 ± 16 bpm vs. SSCS group: 328 ± 11 bpm; *p *= 0.6)

### Effect of vehicle injection into the 4th V

According to Table 1, injection of vehicle (0.9% NaCl) into the 4th V did not affect basal MAP and HR, tachycardic and bradycardic peak, HR range, SBG and PBG in Wistar rats exposed to fresh air.

**Table 1:** Baseline level of mean arterial pressure (MAP) and heart rate (HR), bradycardic and tachycardic peak and sympathetic (SBG) and parasympathetic baroreflex gain (PBG) in Wistar rats exposed to filtered air treated with vehicle into the 4th V

*Variable*	*Control*	*5 minutes*	*15 minutes*	*30 minutes*	*60 minutes*
***MAP (mmHg)***	105 ± 11	106 ± 12	101 ± 15	102 ± 11	107 ± 10
***HR (bpm)***	351 ± 20	342 ± 21	352 ± 19	363 ± 23	363 ± 22
***Bradycardic Peak (bpm)***	225 ± 34	227 ± 24	242 ± 21	238 ± 32	231 ± 22
***Tachycardic Peak (bpm)***	466.2 ± 24	485 ± 14	480 ± 23	470.3 ± 21	465 ± 15
***HR range (bpm)***	242.8 ± 28	246 ± 19	242 ± 11	243 ± 25	245 ± 23
***PBG (bpm × mmHg^-1^)***	-1.9 ± 0.5	-2.43 ± 0.6	-2.43 ± 0.5	-2.2 ± 0.4	-2.3 ± 0.7
***SBG (bpm × mmHg^-1^)***	-3.1 ± 0.5	-3.1 ± 0.7	-2.8 ± 0.8	-2.9 ± 0.2	-1.9 ± 0.4

N = 7. Means ± SDM

**Figure 2:**
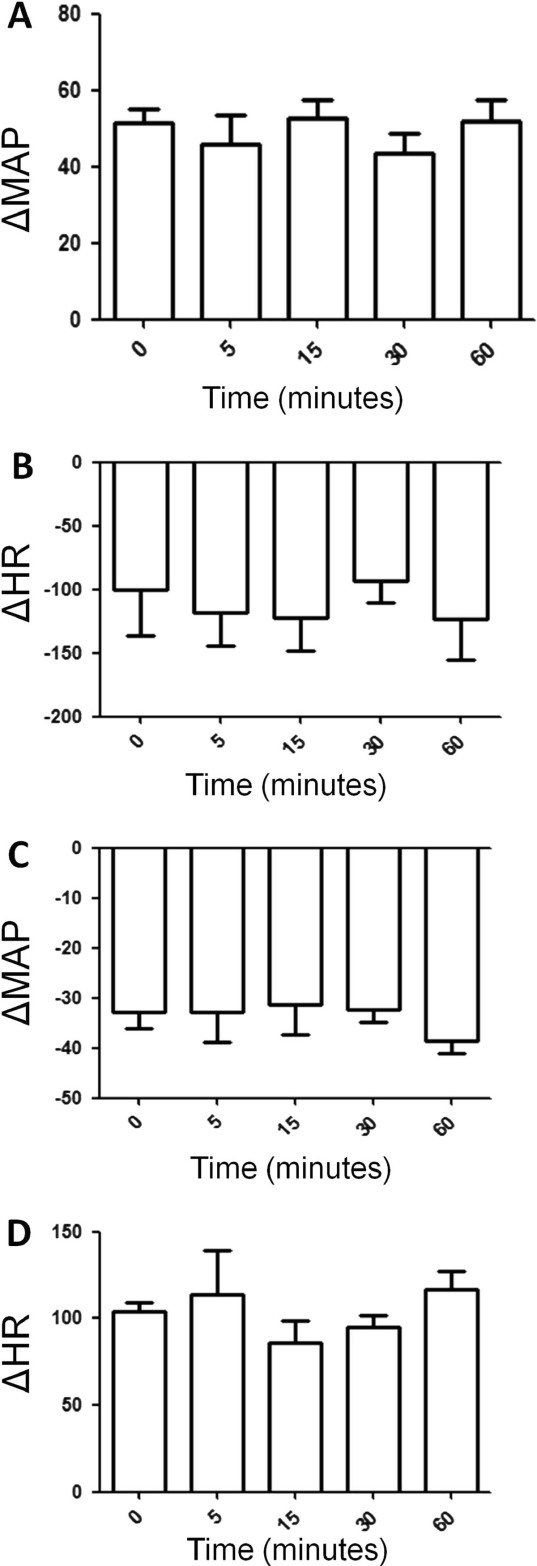
**(A) Increase in mean arterial pressure (MAP, mmHg) and (B) decrease in heart rate (HR, bpm) in response to phenilephrine (PHE, 8 μg/kg i.v., bolus) in Wistar rats exposed to fresh air treated with vehicle into the 4th V**. (**C**) Decrease in mean arterial pressure (MAP, mmHg) and (**D**) increase in heart rate HR, bpm) in response to sodium nitroprusside (SNP, 50 μg/kg i.v., bolus) in Wistar rats exposed to fresh air treated with vehicle into the 4th V. N = 7. Means ± SDM.

Analysis of bradycardic reflex responses to increase in MAP and tachycardic reflex responses to decrease in mean arterial pressure indicated that vehicle injection into the 4th V did not change bradycardic (Figure 2A) and tachycardic reflex (Figure 2B) and increase (Figure 2A) and decrease (Figure 2B) in MAP 5, 15, 30 and 60 minutes after its administration in conscious Wistar rats exposed to fresh air.

We also injected vehicle into the 4th V of rats exposed to SSCS in order to confirm the effects of ATZ. We observed that this treatment did not affect basal MAP and HR, tachycardic and bradycardic peak, HR range, SBG and PBG in Wistar rats exposed to fresh air (Table 2).

**Table 2:** Baseline level of mean arterial pressure (MAP) and heart rate (HR), bradycardic and tachycardic peak and sympathetic (SBG) and parasympathetic baroreflex gain (PBG) in Wistar rats exposed to SSCS treated with vehicle into the 4th V

*Variable*	*Control*	*5 minutes*	*15 minutes*	*30 minutes*	*60 minutes*
***MAP (mmHg)***	101 ± 25	103 ± 11	100 ± 17	107 ± 23	101 ± 11
***HR (bpm)***	365 ± 34	354 ± 24	356 ± 23	367 ± 35	359 ± 19
***Bradycardic Peak (bpm)***	234 ± 23	229 ± 12	232 ± 19	232 ± 21	239 ± 26
***Tachycardic Peak (bpm)***	453 ± 21	475 ± 34	471 ± 15	475 ± 26	481 ± 23
***HR range (bpm)***	248 ± 19	235 ± 23	249 ± 12	239 ± 32	253 ± 25
***PBG (bpm × mmHg^-1^)***	-1.4 ± 0.4	-2.5 ± 0.9	-2.1 ± 0.1	-2.7 ± 0.8	-1.9 ± 0.9
***SBG (bpm × mmHg^-1^)***	-3 ± 0.8	-3.2 ± 0.3	-3 ± 0.9	-2.6 ± 0.1	-2.9 ± 0.9

N = 8. Means ± SDM

**Figure 3:**
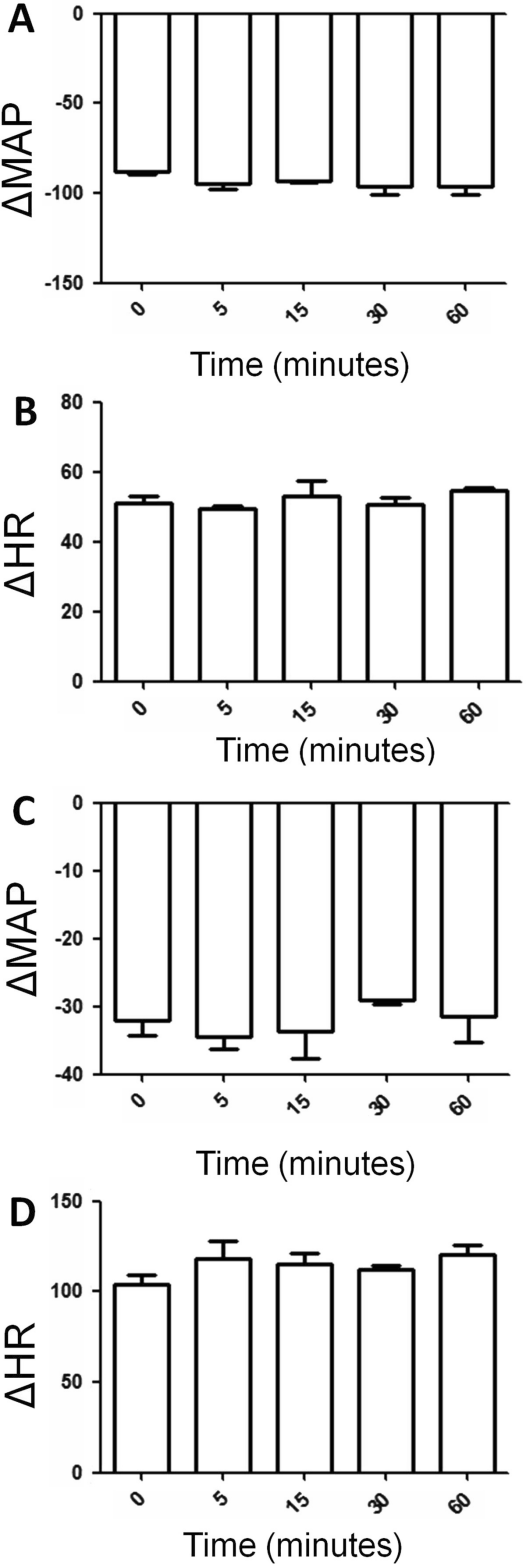
**(A) Increase in mean arterial pressure (MAP, mmHg) and (B) decrease in heart rate (HR, bpm) in response to phenilephrine (PHE, 8 μg/kg i.v., bolus) in Wistar rats exposed to fresh air treated with vehicle into the 4th V**. (**C**) Decrease in mean arterial pressure (MAP, mmHg) and (**D**) increase in heart rate HR, bpm) in response to sodium nitroprusside (SNP, 50 μg/kg i.v., bolus) in Wistar rats exposed to SSCS treated with vehicle into the 4th V. N = 7. Means ± SDM.

Regarding analysis of bradycardic reflex responses to increase in MAP and tachycardic reflex responses to decrease in mean arterial pressure, we noted that vehicle injection into the 4th V did not change bradycardic (Figure 3A) and tachycardic reflex (Figure 3B) and increase (Figure 3A) and decrease (Figure 3B) in MAP 5, 15, 30 and 60 minutes after its administration in conscious Wistar rats exposed to SSCS.

### Effect of ATZ injection into the 4th V

Injections of ATZ into the 4th V did not affect basal MAP, however, it increased basal HR 5 minutes (*p *< 0.05) after its administration in rats exposed to fresh air (Table 3). On the other hand, we did not note significant changes regarding bradycardic and tachycardic peak, HR range, PBG and SBG after central catalase inhibition in rats exposed to fresh air (Table 3).

**Table 3:** Baseline level of mean arterial pressure (MAP) and heart rate (HR), bradycardic and tachycardic peak and sympathetic (SBG) and parasympathetic baroreflex gain (PBG) in Wistar rats exposed to filtered air treated with ATZ into the 4th V

*Variable*	*Control*	*5 minutes*	*15 minutes*	*30 minutes*	*60 minutes*
***MAP (mmHg)***	114 ± 12	119 ± 13	118 ± 11	110 ± 10	111 ± 12
***HR (bpm)***	308 ± 29	394 ± 51*	381 ± 40	327 ± 31	307 ± 25
***Bradycardic Peak (bpm)***	197 ± 32	288 ± 56	269 ± 47	237 ± 43	212 ± 29
***Tachycardic Peak (bpm)***	445 ± 30	475 ± 43	467 ± 30	447 ± 31	451 ± 31
***HR range (bpm)***	243 ± 31	186 ± 32	197 ± 34	220 ± 29	239 ± 28
***PBG (bpm × mmHg^-1^)***	-1.84 ± 0.4	-1.17 ± 0.9	-2.9 ± 0.8	-2.38 ± 0.9	-1.8 ± 0.4
***SBG (bpm × mmHg^-1^)***	-3.59 ± 0.7	-2.69 ± 0.3	-2.33 ± 0.7	-3.38 ± 0.9	-2.93 ± 0.6

N = 8. Means ± SDM. *p < 0.05: Different of Control

**Figure 4:**
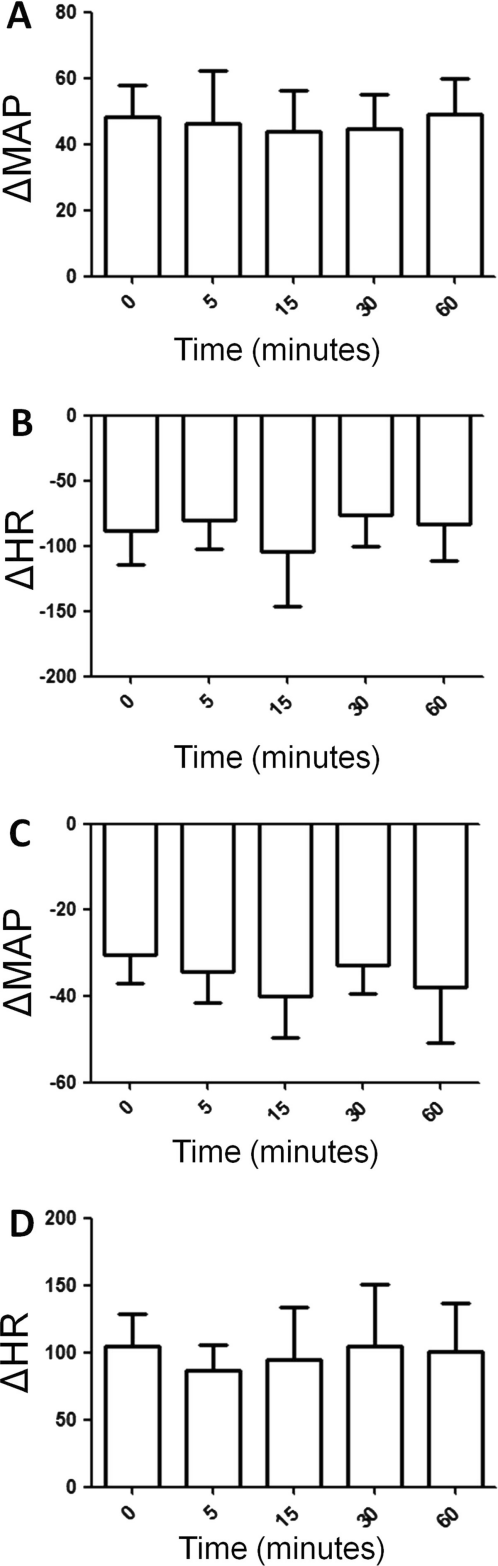
**(A) Increase in mean arterial pressure (MAP, mmHg) and (B) decrease in heart rate (HR, bpm) in response to phenilephrine (PHE, 8 μg/kg i.v., bolus) in Wistar rats exposed to fresh air treated with ATZ into the 4th V**. (**C**) Decrease in mean arterial pressure (MAP, mmHg) and (D) increase in heart rate (HR, bpm) in response to sodium nitroprusside (SNP, 50 μg/kg i.v., bolus) in Wistar rats exposed to fresh air treated with ATZ into the 4th V. N = 8. Means ± SDM.

Based on Figure 4A, ATZ injection into the 4th V did not change bradycardic reflex response to PHE-induced increase in arterial pressure and increase in MAP in rats exposed to fresh air. In addition, tachycardic reflex responses to SNP and SNP-induced decrease in MAP were not influenced by catalase inhibition into the 4th V in rats exposed to fresh air (Figure 4B).

In relation to the group exposed to SSCS we observed that catalase inhibition into the 4th V increased basal HR during the first 15 minutes after ATZ administration (*p *< 0.05) without affect basal MAP (Table 4). Furthermore, bradycardic peak was also attenuated during the first 15 minutes after catalase inhibition into the 4th V (*p *< 0.05). On the other hand, there were no changes with respect to tachycardic peak, HR range, PBG and SBG after ATZ treatment in Wistar rats exposed to SSCS (Table 4).

**Table 4:** Baseline level of mean arterial pressure (MAP) and heart rate (HR), bradycardic and tachycardic peak and sympathetic (SBG) and parasympathetic baroreflex gain (PBG) in Wistar rats exposed to SSCS treated with ATZ into the 4th V

*Variable*	*Control*	*5 minutes*	*15 minutes*	*30 minutes*	*60 minutes*
***MAP (mmHg)***	109 ± 11	114 ± 16	113 ± 12	109 ± 15	108 ± 12
***HR (bpm)***	328 ± 26	418 ± 37*	420 ± 38*	387 ± 29	346 ± 19
***Bradycardic Peak (bpm)***	230 ± 29	322 ± 42*	306 ± 23*	265 ± 14	253 ± 19
***Tachycardic Peak (bpm)***	498 ± 23	520 ± 23	516 ± 19	516 ± 20	477 ± 21
***HR range (bpm)***	261 ± 22	198 ± 41	210 ± 22	250 ± 14	224 ± 23
***PBG (bpm × mmHg^-1^)***	-2.17 ± 0.4	-2.24 ± 0.7	-3.07 ± 0.9	-2.58 ± 0.6	-1.68 ± 0.4
***SBG (bpm × mmHg^-1^)***	-2.95 ± 0.4	-2.08 ± 0.7	-2.64 ± 0.8	-2.68 ± 0.9	-3.07 ± 0.6

N = 7. Means ± SDM. *p < 0.05: Different of Control

**Figure 5:**
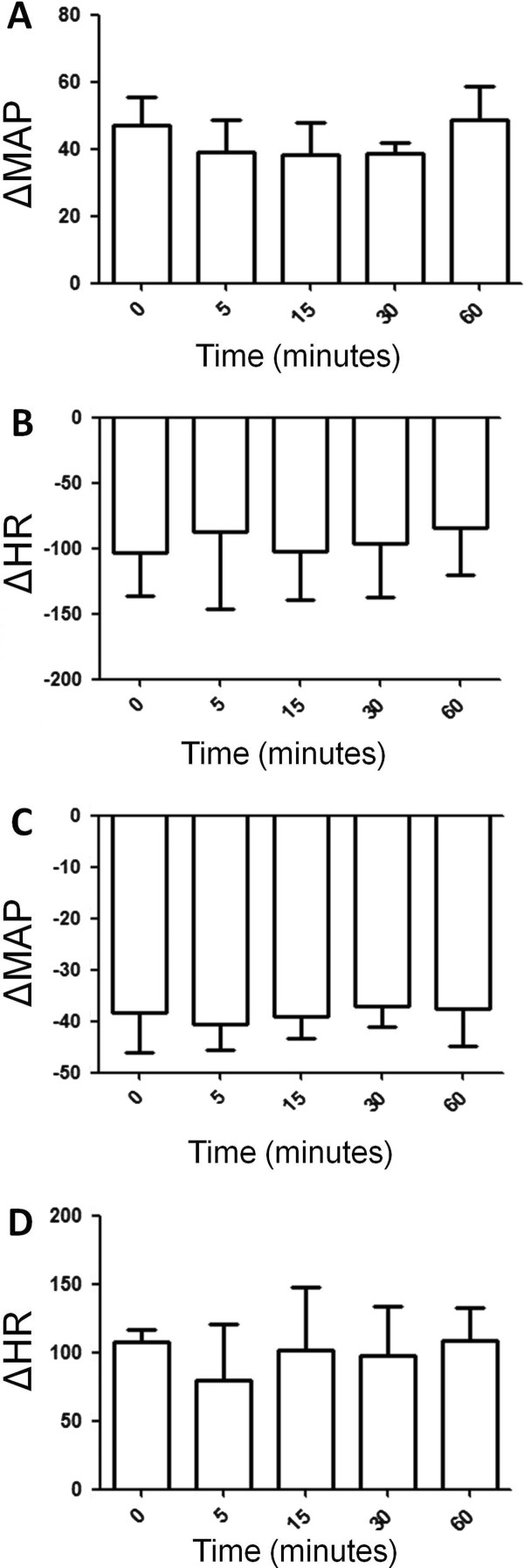
**(A) Increase in mean arterial pressure (MAP, mmHg) and (B) decrease in heart rate (HR, bpm) in response to phenilephrine (PHE, 8 μg/kg i.v., bolus) in Wistar rats exposed to SSCS treated with ATZ into the 4th V. N = 7**. (**C**) Decrease in mean arterial pressure (MAP, mmHg) and (**D**) increase in heart rate (HR, bpm) in response to sodium nitroprusside (SNP, 50 μg/kg i.v., bolus) in Wistar rats exposed to SSCS treated with ATZ into the 4th V. N = 7. Means ± SDM.

According to Figure 5A, injection of ATZ into the 4th V did not change bradycardic reflex response to PHE-induced increase in arterial pressure and increase in MAP in rats exposed to SSCS. Additionally, tachycardic reflex responses to SNP and SNP-induced decrease in MAP were not influenced by central catalase inhibition in rats exposed to SSCS (Figure 5B).

## Discussion

In this investigation we endeavored to evaluate the effects of catalase inhibition into the 4th V on baroreflex and cardiovascular responses in rats exposed to SSCS. We reported that catalase inhibition increased basal HR during the first 5 minutes in rats exposed to fresh air while it increased basal HR and attenuated bradycardic peak during the first 15 minutes in rats exposed to SSCS. We suggest that SSCS exposure increased the cardiovascular responses caused by catalase inhibition into the 4th V. The lack of any change in the vehicle group is consistent with this assumption.

### SSCS effects on basal HR and MAP

There was no significant change on basal MAP and HR between rats exposed to SSCS and fresh air. Those findings are in accordance with previous studies from our group, which evaluated the effects of SSCS (three weeks) on basal MAP and HR in Wistar [23], Wistar Kyoto (WKY) rats and spontaneously hypertensive rats (SHR) [24]. We choose to use this period of SSCS exposure in order to investigate if this protocol is able to influence the neural control of the cardiovascular system without affect basal MAP and HR. We may hypothesize that higher levels of exposure to SSCS would affect with more intensity cardiovascular function. Nevertheless, a recent study suggested that acute inhalation exposure to concentrated air particulate elevates blood pressure in chronically instrumented dogs [21]. Several reasons may explain the difference between this investigation and our findings. First, while they exposed dogs to fine particles (diameters between 0.15 and 2.5 μm) we exposed rats to SSCS, which contains thousands of toxic chemical components known and particles with higher diameter than 2.5 μm [29]. However, we did not measure the diameters of the particles. Second, blood pressure was evaluated through telemetry and we cannulated femoral artery in order to more accurate measure arterial pressure. Third, they exposed canine models repeatedly for a single period and in our study rats were exposed during three weeks. Fourth, in our research rats were exposed 180 minutes per day, and Bartoli et al. [21] investigation exposed animals to concentrated air particle single 5-hour period. Even though we consider all those differences regarding methodological aspects, we believe that three weeks of exposure to SSCS was not enough to cause the same effects observed in the study of Bartoli et al. [21].

### SSCS effects on baroreflex

The tachycardic peak represents the maximal sympathetic response to decrease in arterial pressure; the bradycardic peak is an index that indicate the highest parasympathetic response to elevation in blood pressure; the HR range index corresponds to the difference between the upper and lower HR peak and the derivation of HR in function of MAP variation is an index of baroreflex gain [30]. According to our data catalase inhibition into the 4th V attenuated bradycardic peak during the first 15 minutes in rats exposed to SSCS while it was not affected in rats exposed to fresh air. This finding indicates that SSCS exposure affects the parasympathetic activity in Wistar rats. A recent study demonstrated that vagal modulation of the heart is blunted in heavy smokers, particularly during a parasympathetic maneuver [31]. Moreover, Luchese et al. [20] demonstrated that acute cigarette smoke exposure increases oxidative stress in the brain. However, they did not investigate which area of the brain was affected. Taken together, we suggest that increased oxidative stress in areas surrounding the 4th V caused by SSCS exposure is involved in this mechanism, since those areas such as nucleus ambiguus, the area postrema of the NTS and dorsal motor nucleus of the vagus are related to the parasympathetic activity [19].

In our research the baroreflex was evaluated in conscious rats, since baroreflex is blunted under anesthesia [32] reducing the range of HR, which outcomes in an analysis of a restricted portion of the baroreflex response. Therefore, we believe that our investigation provides trustful information regarding the effects of the catalase inhibitor ATZ into the 4th V on baroreflex components in conscious Wistar rats. These data present relevant information, since currently baroceptor reflex is largely studied in different models and strain of rats [33,34] aiming to prevent hypertension development, due to the fact that reduced baroreflex function is indicative of cardiovascular disease [35-37].

### SSCS effects on brain oxidative stress and cardiovascular responses

In this study we aimed to verify the effects of catalase inhibition into the 4th V on cardiovascular responses in rats exposed to SSCS. Catalase is a common enzyme found in nearly all living organisms that are exposed to oxygen, where it catalyzes the decomposition of hydrogen peroxide to water and oxygen [8]. A previous study observed that exogenous H_2_O_2 _into the 4th V affects the neural regulation of the cardiovascular system [38]. We choose this enzyme because we aimed to verify if endogenous H_2_O_2 _is involved in this process.

We noted that acute ATZ injected into the 4th V did not change basal HR and MAP in conscious Wistar rats. Cardoso et al. [38] have previously indicated that exogenous H_2_O_2 _injected into the 4th V of conscious Wistar rats caused bradycardic and hypertensive dose-dependent responses. Furthermore, those bradycardic responses were abolished by intravenous atropine, hypertensive responses were abolished by central catalase and N-acetylcystein treatment as well as with intravenous prasozin. Another study of Cardoso et al. [39] showed that H_2_O_2 _into the NTS induces hypotension and bradycardia. Considering that ATZ is a catalase inhibitor and it consequently increases endogenous H_2_O_2 _[8] we expected that ATZ into the 4th V would cause the same responses observed in the research of Cardoso et al. [38]. If we compare their report with our data there are some points that should be addressed such as the H_2_O_2 _source, in our study we investigated endogenous H_2_O_2 _by catalase inhibition, in Cardoso et al. investigation they used exogenous H_2_O_2 _[38].

We observed that acute endogenous H_2_O_2 _increase in the 4th V caused by catalase inhibition affected the parasympathetic regulation of the cardiovascular system, since it increased basal HR with a higher intensity in rats exposed to SSCS and reduced bradycardic peak in animals exposed to SSCS with no alteration in rats exposed to fresh air. The nose anatomy is well suitable for the transport of exogenous agents into the brain. Some xenobiotics, i.e., viruses, can enter the brain via several cranial nerves, on the other hand, the olfactory nerve is only vulnerable to such penetration. The dendritic knobs and protruding cilia of the olfactory receptor cells that make up this nerve present an exposed surface area conservatively estimated at 23 cm [40]. Those cells are extensively distributed all through the rostral part of the nasal cavity, embedded in a specialized neuroepithelium that lines the region of the cribriform plate, the dorsal septum, and sectors of the superior and middle turbinates. Distinct from other receptor cells, those cells are first-order neurons, which projects axons directly to the brain without intervening synapse. Therefore, we believe that the olfactory vector hypothesis for neurological diseases may explain our data [41]. Nonetheless, we can not confirm which area was affected by SSCS exposure. In view of the anatomical scope of the 4th V, an action on an only one neuronal cluster is not an easy accomplishment, however, prior researches indicated a preference for parasympathetic system which modulates HR, such as the dorsal motor nucleus of the vagus and nucleus ambiguus, that receive glutamathergic projections from the nucleus of the solitary tract [18]. We suggest future studies to investigate which areas are affected by SSCS.

### SSCS versus mainstream cigarette smoke

We used SSCS exposure in this investigation since it has been inferred that SSCS is more toxic than mainstream cigarette smoke [42]. The Philip Morris Co privately performed extensive in vivo toxicological testing of sidestream smoke at its secret Institut für Biologische Forschung (INBIFO) in Germany [42]. There are several published bacterial mutagenesis studies indicating that sidestream particulate matter and condensates are more mutagenic than mainstream particulate matter and condensates [43,44]. The protocol which the authors investigated bacterial mutagenesis is recognized as a risk for humans [44-46]. Sidestream smoke contains between 15-300 times more ammonia than mainstream smoke. Apart from nicotine, cigarette smoke contains thousands of other chemical substances, including carbon monoxide, hydrogen cyanide, nitrogen oxides, aldehydes, N-nitrosamines, polyaromatic hydrocarbons. SSCS has a higher concentration of toxic substances compared to mainstream smoke due to a lower temperature of combustion as well as lack of filtering [43]. For instance, there is five times more acrolein in SSCS compared to mainstream smoke [47]. By inhalation, whole fresh sidestream smoke is 2-6 times more toxic per gram than mainstream smoke, depending on the end point [29]. Our results and those information are relevant to direct and implement public politics and improve public health system.

### Limitations

Our investigation presents some points that should be addressed. 1) we did not measure the concentration of H_2_O_2 _or other ROS inside the 4th V before and after the injection of ATZ. It would significantly strengthen the impact of our results to show that H_2_O_2 _or some other ROS is actually altered in the 4th V with this duration and level of treatment. Mao and coworkers [48] have already demonstrated a method that performed a continuous on-line measurement of cerebral H_2_O_2 _using enzyme-modified ring-disk plastic carbon film electrode. Unfortunately, we did not measure endogenous H_2_O_2 _due to the lack of such equipment in our laboratory. On the other hand, our method is well accepted in the literature, since previous studies observed significant association between antioxidant injections and antioxidant activity into the brainstem [13,49]. We suggest future studies to perform such method; 2) the last exposure to cigarette smoke occurred 5 days prior to cardiovascular testing and data collection due to our surgery protocols. Oxidative stress may occur rapidly and reactive oxygen species dissipate quickly. On the other hand, based on our pilot studies, rats do not stand SSCS exposure after cerebral surgery. In order to address to what extent the surgery may contribute to the creation of ROS and other harmful metabolites, another indirect indication would be to determine the number of apoptotic cells in this region of the brain (e.g. Caspase-3 staining) and the amount of fibrotic tissue (e.g. Masson's trichrome staining). Future protocols could investigate this mechanism through those procedures.

## Conclusion

Our findings indicate that catalase inhibition into the 4th V has stronger effects on rats exposed to SSCS. We suggest that SSCS exposure increase oxidative stress in brainstem areas that regulate cardiovascular system.

## Abbreviations

SSCS: Sidestream cigarette smoke; ROS: Reactive oxygen species; H_2_O_2_: Hydrogen peroxide; SOD: Superoxide dismutase; NTS: Nucleus of the solitary tract; CVLM: Caudal ventrolateral medulla; RVLM: Rostral ventrolateral medulla; 4th V: Fourth cerebral ventricle; CO: Carbon monoxide; MAP: Mean arterial pressure; HR: Hear rate; SBG: Sympathetic baroreflex gain; PBG: Parasympathetic baroreflex gain; PHE: Phenylephrine; SNP: Sodium nitroprusside; ATZ: 3-Amino-1,2,4-triazole; WKY: Wistar Kyoto rats; SHR: Spontaneously hypertensive rats.

## Competing interests

The authors declare that they have no competing interests.

## Authors' contributions

VEV, LCA, MAS, CF, FA, FLAF, VX, MG, CBM, LCMV and PHNS participated in the acquisition of data and revision of the manuscript. All authors conceived of the study, determined the design, performed the statistical analysis, interpreted the data and drafted the manuscript. All authors read and gave final approval for the version submitted for publication.

## Pre-publication history

The pre-publication history for this paper can be accessed here:

http://www.biomedcentral.com/1471-2261/12/22/prepub
